# Key role of scale morphology in flatfishes (Pleuronectiformes) in the ability to keep sand

**DOI:** 10.1038/srep26308

**Published:** 2016-05-20

**Authors:** Marlene Spinner, Mareike Kortmann, Camille Traini, Stanislav N. Gorb

**Affiliations:** 1 Zoological Institute, Functional Morphology and Biomechanics, Kiel University, Am Botanischen Garten 9, 24118 Kiel, Germany; 2 Department of Sedimentology, Kiel University, Otto-Hahn-Platz 1, 24118 Kiel, Germany

## Abstract

Flatfishes bury themselves for camouflage and protection. Whereas species-specific preferences for certain sediments were previously shown, the role of scales in interaction with sediment has not been investigated. Here, scale morphology and sediment friction were examined in four European pleuronectiforms: *Limanda limanda, Platichthys flesus, Pleuronectes platessa*, and *Solea solea*. All species had different scale types ranging from cycloid to ctenoid scales. On the blind side, the number of scales is higher and scales have less ctenial spines than on the eye side. The critical angle of sediment sliding (static friction) significantly depended on the grain size and was considerably higher on the eye side. The effect of mucus was excluded by repeated measurements on resin replicas of the skin. Our results demonstrate the impact of scale morphology on sediment interaction and give an insight about the ability of scales to keep sand. Exposed scales and a higher number of ctenial spines on the eye side lead to an increase of friction forces, especially for sediments with a smaller grain size. Our results suggest that the evolution of scales was at least partly driven by their interactions with sediment which confirms the relevance of sediment for the distribution and radiation of Pleuronectiformes.

The scales of flatfishes (Pleuronectiformes) are highly variable and differ in both their shape and arrangement. Some species have juxtaposed cycloid scales embedded in the underlying tissues, whereas the skin of others is covered by overlapping ctenoid scales^1^,^2^,^3^. Also tubercles or granular plates can be found^1^. Although the impact of the sediment type on behaviour and population density demonstrate the relevance of the sediment for numerous flatfish species, the interaction of the different scale types with different types of sediments has never been investigated in this group.

Flatfishes are ground dwelling and burying animals. In their development, juveniles lay down on one side on the ground while one eye migrates to the upper side of the body (eye side), whereas the lower side is called blind side. As a result, individuals have two sides with very different functions. Whereas both sides are involved in burying activity and the interactions with sediment, the upper side has adapted colors for camouflage and is often additionally covered with sediment.

Numerous behavioural studies showed that individual flatfish species have a clear preference for a certain grain size and material of sediments^4^,^5^,^6^,^7^,^8^. Field studies clearly demonstrated a dependency of the abundance of certain flatfish species on the presence of sediments of specific grain size^6^,^9^. Although the selectivity differs among species^7^, there is a trend towards larger grain size with increasing body length^5^,^6^: adult animals prefer coarser sediments than the juveniles^4^,^10^. The ability to bury in the sediment seems to be a decisive factor for flatfish site selection^7^, and sediments preventing burying ability were avoided^6^.

The extraordinary ability of flatfishes, to adapt the coloration pattern of their eye side to a wide range of substrates, has been shown in many species^11^,^12^,^13^,^14^. In addition to their camouflaging skin coloration, many species bury themselves or have a thin coverage of sand on their eye side, while resting on the sea floor. A study on camouflage and burying activity of *Parophrys vetulus, Lepidopsetta polyxystra*, and *Hippoglossus stenolepis* revealed the importance of this behaviour^14^. In case of longer adaptation period (which can take one day), a sand coverage is generated by the fish to compensate the mismatching of camouflage^14^. It can be assumed that different species specific scale types on the eye side and the blind side are the result of adaptation to burying due to the frequent contact with the sediment, as well as to preventing removal of the sand coverage from the skin by water currents.

In this study we investigated the sliding properties of sediments of different grain size on the skin of the four flatfish species *Limanda limanda, Platichthys flesus, Pleuronectes platessa*, and *Solea solea* to shed light on the scale interaction with various sediments. The species studied represent the high variability of scale types among Pleuronectiformes ranging from embedded cycloid scales (*P. platessa*) to exposed ctenoid (*L. limanda* and *S. solea*) and tubercular scales (*P. flesus*). Aiming at testing possible differences in frictional properties between the eye and blind side, as well as between sediment types, our functional study gives an insight into selective factors behind the evolution of the wide range of scale types. The scale types can differ in closely related species and between the eye- and blind side^1^. It can change even in a single individual during its life span^1^,^3^,^15^. In order to eliminate the influence of differences in both skin and scale materials we additionally studied critical sliding angles on epoxy resin replicas of fish skin. This also excludes the effect of mucus that might influence the frictional and hydrodynamical properties^16^,^17^,^18^.

## Results

### Scale morphology and density

**Figure 1:**
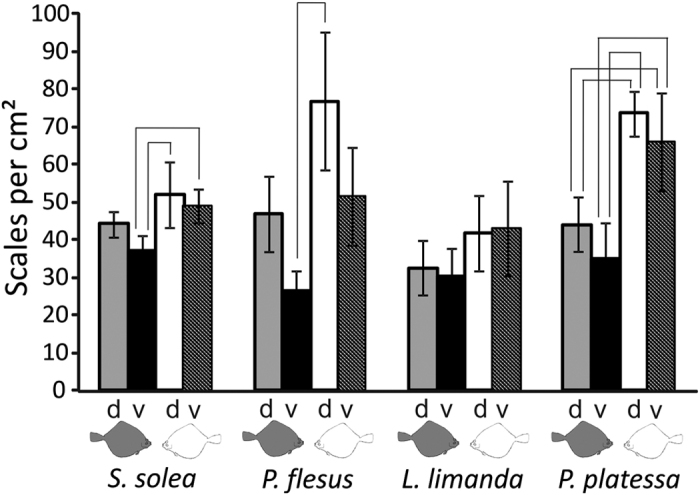
Scale density of *S. solea, P. flesus, L. limanda*, and *P. platessa.* Scale density on the dorsal (d) and ventral (v) part of the eye side (gray fish symbol) and blind side (white fish symbol). Standard deviation is presented by error bars. Brackets indicate significant differences (One-Way ANOVA, P ≤ 0.001; multiple comparison procedures Holm-Sidak method, P < 0.05).

Morphological features present in all individuals of one species, but absent in the three others, provide a robust view on species-specific scale morphology in Pleuronectiformes. Our data showed a difference between the eye side and the blind side in all species studied (Fig. 1) (One-Way ANOVA, P ≤ 0.001; multiple comparison procedures Holm-Sidak method, P < 0.05). The highest density of scales was found on the blind side of *P. platessa* (Fig. 1). The difference between the eye side and the blind side was higher for the species with cycloid scales (*P. platessa* and *P. flesus*) (Fig. 1). *L. limanda* had a difference between the eye side and the blind side. However, the trend was not great enough to be statistically significant (Fig. 1).

**Figure 2:**
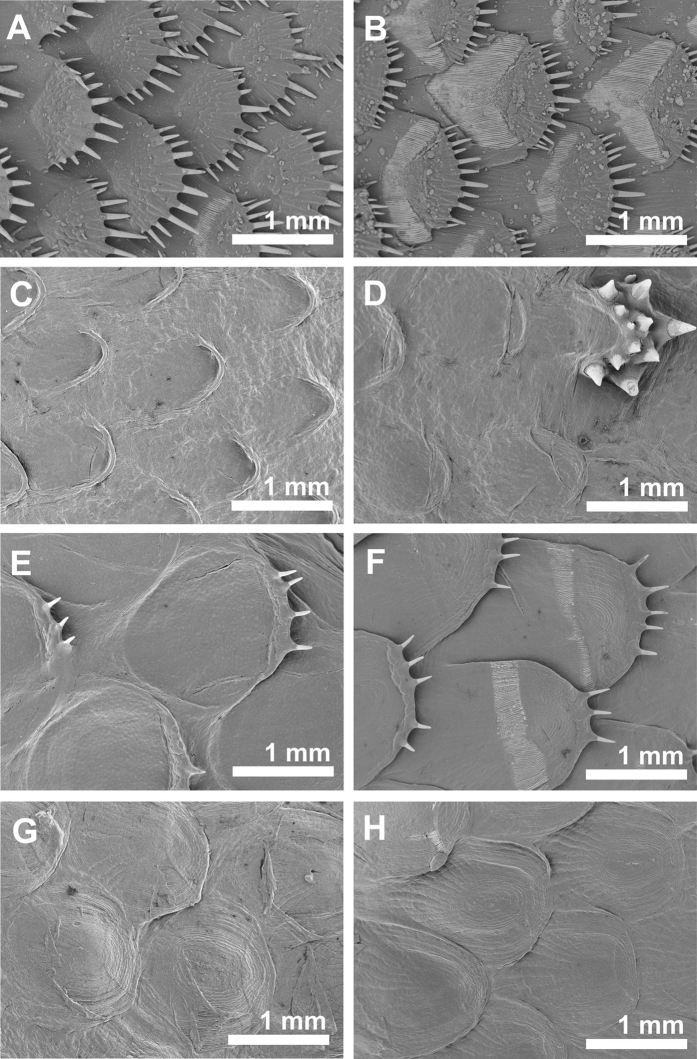
Scanning electron micrographs of scales of Pleuronectiformes. (**A**) Scales of the blind side of *S. solea*. (**B**) Scales of the eye side of *S. solea.* (**C**) Scales of the blind side of *P. flesus*. (**D**) Scales of the eye side of *P. flesus.* (**E**) Scales of the blind side of *L. limanda*. (**F**) Scales of the eye side of *L. limanda.* (**G**) Scales of the blind side of *P. platessa*. (**H**) Scales of the eye side of *P. platessa.*

**Figure 3:**
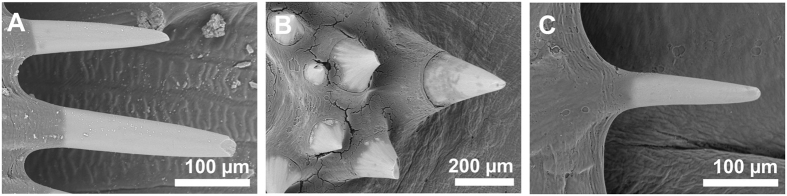
Scanning electron micrographs of ctenial spines of scales of Pleurenectiformes. (**A**) *S. solea*. (**B**) *P. flesus.* (**C**) *L. limanda.*

The scale morphology varied greatly across the species but was consistent in individuals of the same species and was in accordance to the description in taxonomic keys (see Norman^1^). *S. solea* had strongly overlapping ctenoid scales of 1.5 mm diameter on both sides of the body. Scales have 11–12 ctenial spines on the eye side and 11–15 ctenial spines on the blind side (Figs 2A,B and 3A). In *P. flesus* we found the largest differences between scales on the eye side and the blind side. In addition to deep embedded cycloid scales, the eye side was covered by tuberculate scales having 9–14 ctenial spines (Fig. 2C,D). Spines were arranged in two or three semicircles nearly perpendicular to the scale surface at the distal part of the scale (Fig. 2D). Toward the scale edges the length of the spinies increased from 50 to 200 μm (Figs 2D and 3B). On the blind side ctenoid scales were only present along the lateral line, around the gill cover, and along the dorsal and ventral fin, but with great variation between the individuals, whereas the rest of the body was covered by cycloid scales. Irrespective of the presence or absence of spines, scales overlapped each other only slightly and had a diameter of about 0.8–1.0 mm on the whole body of the fish (Fig. 2C,D). All individuals of *L. limanda* had exposed ctenoid scales of a diameter of 2 mm on both sides having 2–4 ctenial spines per scale on the blind side (Fig. 2E) and 3–11 ctenial spines per scale on the eye side (Fig. 2F). The spines had a length of about 200 μm and a thickness of about 50 μm with decreasing size towards the scale border (Fig. 3C). Scales on the blind side were more circular and less overlapping than scales of the eye side (Fig. 2E,F). Individuals of *P. platessa* had overlapping deep-embedded cycloid scales of 1.0 –1.2 mm diameter on both sides of the body (Fig. 2G,H).

### Critical angle of sediment sliding on the fish skin

**Figure 4:**
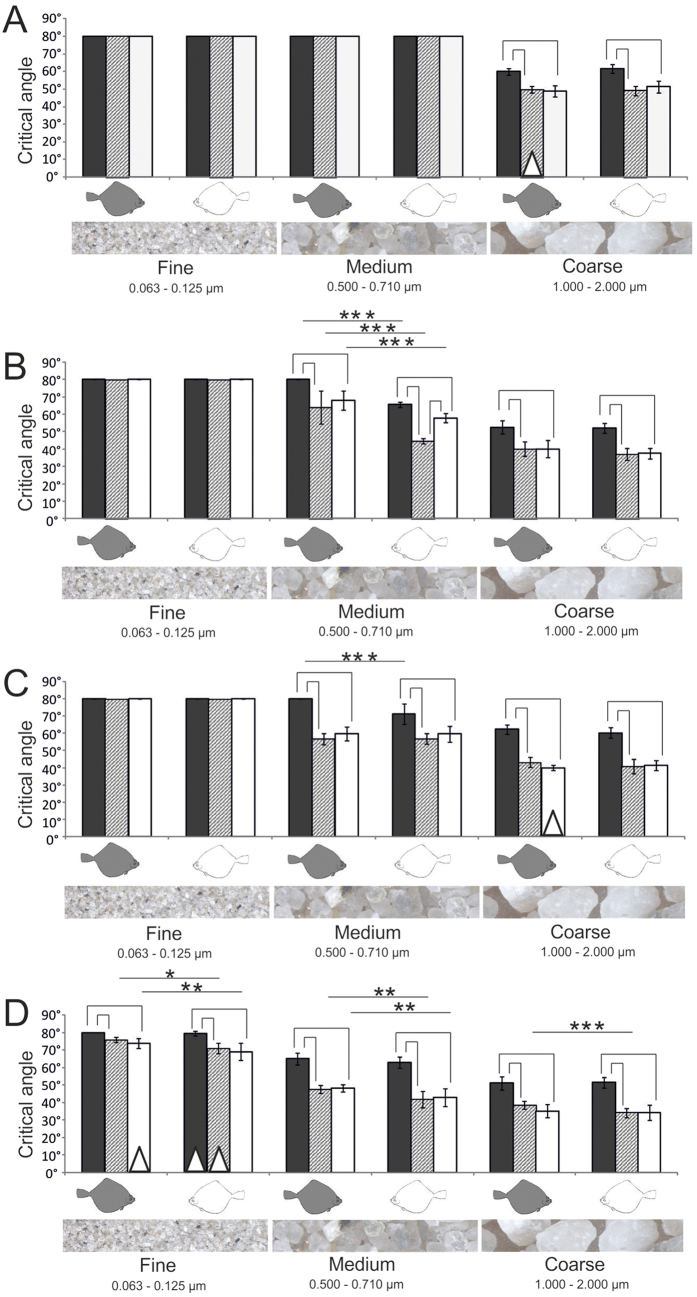
CSAs of the three sediment types (fine, medium, and coarse) in three directions: cranial (black bars), caudal (gray bars), and lateral (white bars) on the eye side (gray fish symbol) and blind side (white fish symbol). (**A**) *S. solea* (**B**) *P. flesus*. (**C**) *L. limanda*. (**D**) *P. platessa*. Brackets indicate significant differences of CSAs of the different sliding directions (Kruskal-Wallis One-Way ANOVA on Ranks, P ≤ 0.001, Tukey test P < 0.05). Lines indicate significant differences between the eye side and the blind side (Mann-Whitney Rank Sum test: *P ≤ 0.05, **P ≤ 0.01, ***P ≤ 0.001). Standard deviation is presented by error bars. Triangles indicate measurements that were not normally distributed. Data are presented separately as box plots in the supplementary material (S2).

**Figure 5:**
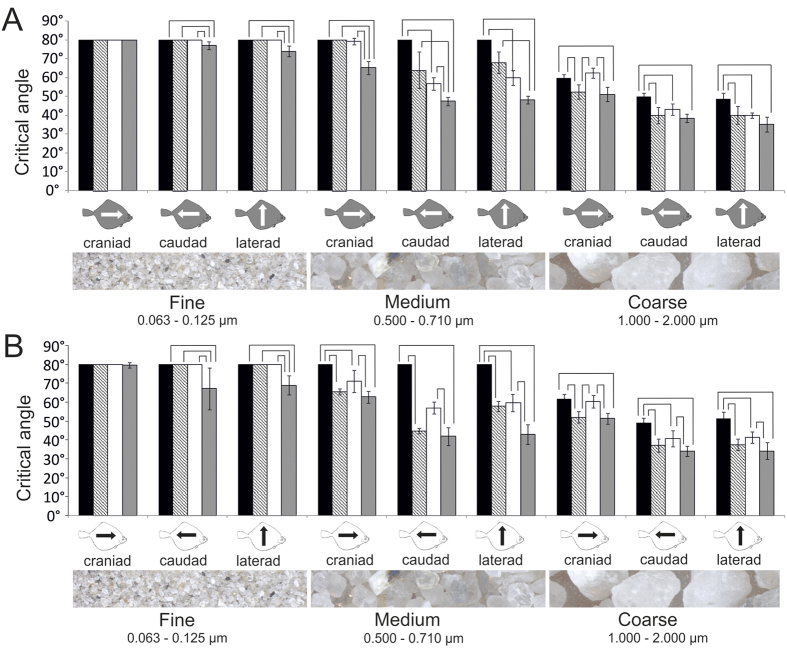
CSAs of the three sediment types (fine, medium, and coarse) in three directions: cranial, caudal, and lateral on skin of *S. solea* (black bars), *P. flesus* (hatched bars), *L. limanda* (white bars), and *P. platessa* (gray bars). (**A**) Skin of the eye side. (**B**) Skin of the blind side. Brackets indicate significant differences of CSAs in different sliding directions (Kruskal-Wallis One-Way ANOVA on Ranks, 3 DF, P ≤ 0.001; Dunn´s method, P < 0.05). Standard deviation is presented by error bars.

**Figure 6:**
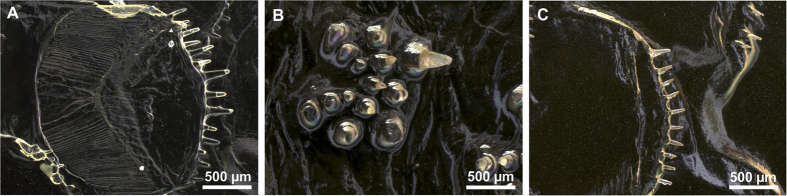
Light microscopy images of replicas of flat fish skin. (**A**) Ctenoid scale of the blind side of *S. solea*. (**B**) Tubercle scale of the eye side of *P. flesus*. (**C**) Ctenoid scale of the eye side of *L. limanda*.

**Figure 7:**
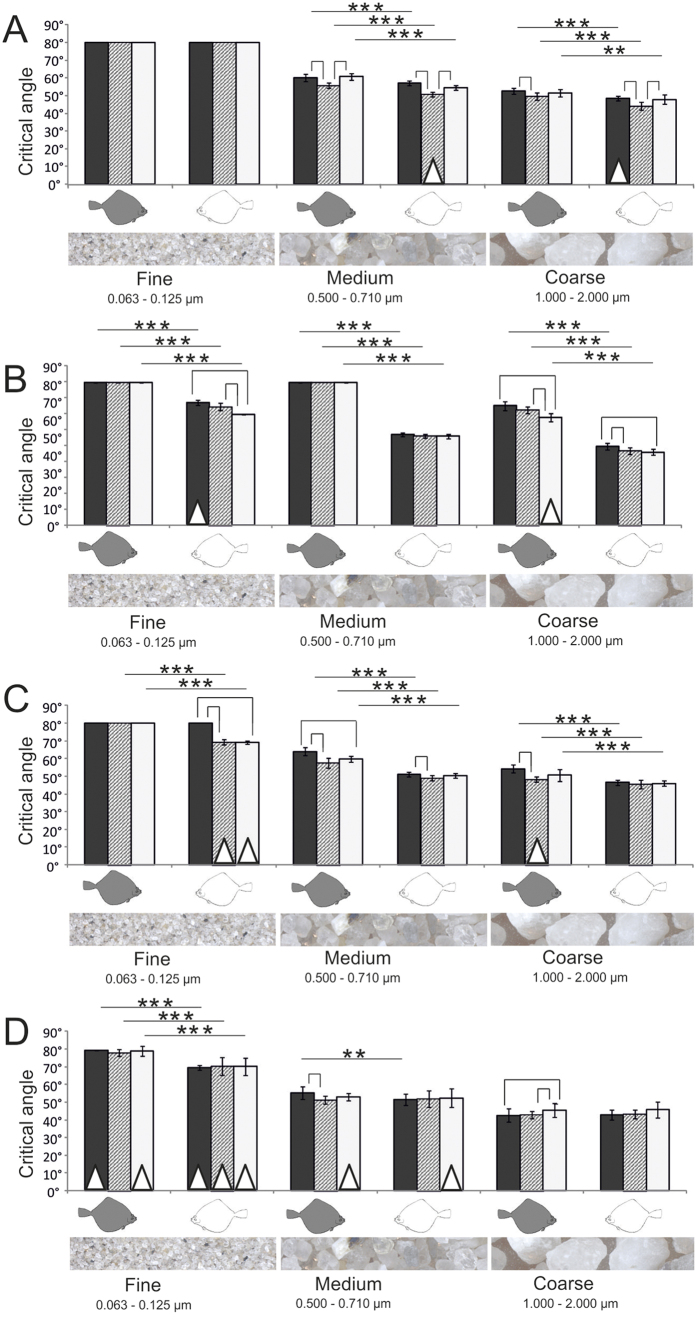
CSAs of the three sediment types (fine, medium, and coarse) in three directions: cranial (black bars), caudal (gray bars), and lateral (white bars) on resin replicas of the eye side (gray fish symbol) and blind side (white fish symbol). (**A**) *S. solea*. (**B**) *P. flesus*. (**C**) *L. limanda*. (**D**) *P. platessa*. Brackets indicate significant differences of CSAs of the different sliding directions (Kruskal-Wallis One-Way ANOVA on Ranks, P ≤ 0.001, Tukey test P < 0.05). Lines indicated significant differences between the eye side and the blind side (*P ≤ 0.05, **P ≤ 0.01, ***P ≤ 0.001). Standard deviation is presented by error bars. Triangles indicate measurements that were not normally distributed. Data are presented separately as box plots in the supplementary material (S2).

The critical angle of sediment sliding (CSA) was species-specific, direction-dependent, and significantly different for the three sediment types. The experimental setup measuring a sliding event benefited a skewness with overrepresentation of certain angles and was below the range of the CSAs especially in *S. solea* and for fine sediments. This is reflected by the distribution of data, of which only 56% were normally distributed (Kolmogorov-Smirnov-Test) and 24% had only the maximum value for the sliding angle of 80°. In the following we therefore present mean values in our figures (Figs 4, 5, 6, 7) and analyzed the data using more powerful non-parametric statistics. Measurements that were not normally distributed are presented as box plots in the supplementary material (S2).

Except for the finest sediment in *S. solea, L. limanda*, and *P. platessa*, and the sediment of medium grain size in *S. solea*, the CSA was significantly higher in the rostral (against the scales) direction than in both lateral and caudal directions (Kruskal-Wallis One-Way ANOVA on ranks, P ≤ 0.001; pairwise multiple comparison Tukey test, P < 0.05; H and q-values see supplementary material S3).

The CSAs were significantly different for different sediment types. Grains of larger size slid down at lower inclination than the finer sediments (Kruskal-Wallis One-Way ANOVA on ranks, 2 DF, P ≤ 0.001; pairwise multiple comparison Tukey test, P < 0.05; H and q-values see S4). Since the sand was placed as very thin layer, interactions within the sediment volume (between sediment layers) can be largely excluded as cause for the differences in sliding behavior. One exception of this trend was the skin at both body sides of *S. solea* which generated also for the medium sediment as high friction as for the fine sediment (Kruskal-Wallis One-Way ANOVA on ranks, 2 DF, P ≤ 0.001; pairwise multiple comparison Tukey test, P < 0.05). This was also the case for the skin of the eye side of *L. limanda* (Kruskal-Wallis One-Way ANOVA on ranks, 2 DF, P ≤ 0.001; pairwise multiple comparison Tukey test, P < 0.05).

With an exception of the fine sediment in craniad direction, all measurements revealed species-specific differences particularly remarkable for *S. solea* and *P. platessa* (Fig. 5). The following comparisons are the result of a Kruskal-Wallis One-Way ANOVA on Ranks (3 DF, P ≤ 0.001) and a subsequent pairwise comparison test (Dunn’s method, P < 0.05). Significant differences are presented in Fig. 5. Sliding measurements in lateral and caudal direction revealed a significantly lower friction for the fine sediment in *P. platessa* than in all the other species studied. This applied for both the eye and the blind side. Also for medium sediment, measured on the eye side in both cranial and caudal directions, *P. platessa* showed the lowest CSAs. In the lateral direction the CSA was lower than in all other species, but significantly different only for *S. solea* and *P. flesus*, since CSAs were also low in *L. limanda* which differed significantly from *S. solea* in both caudal and lateral directions. By contrast, on the blind side of *P. platessa* we found significantly lower angles than in both *S. solea* and *L. limanda,* but not in comparison to *P. flesus*. In *S. solea* the CSAs of the medium and coarse grain size were in general higher than in the other species examined. This trend was statistically significant at the eye side for the medium grain size in rostral direction only in comparison to *P. platessa*, and in both caudad and laterad directions additionally also in *L. limanda,* and at the blind side for all species and directions, except for *L. limanda* in caudad direction. In experiments at both body sides in both caudal and lateral directions in *S. solea,* coarse sediments slid down at significantly higher angles than in all other species studied. In cranial direction the difference was at least significant in comparison to both *P. flesus* and *P. platessa*. The CSAs for the coarse sediment were the lowest in both *P. platessa* and *P. flesus*. We found no statistical difference between these species.

The differences between the eye and the blind side were most evident for the medium grain size (Fig. 4). For this sediment we found significant differences in CSAs in caudal and lateral direction in *P. flesus* (P ≤ 0.01), craniad in *P. platessa* (P ≤ 0.001), and towards all directions in *L. limanda* (P ≤ 0.001) (Mann-Whitney Rank Sum test). In *P. flesus* CSAs were also significantly different towards both caudal and lateral directions for the finest sediment (P ≤ 0.05 and P ≤ 0.01) and in the caudal direction for the coarse grain size (P ≤ 0.001) (Mann-Whitney Rank Sum test). In *S. solea* we found no significant differences between the eye side and the blind side (Mann-Whitney Rank Sum test).

### Critical angle of sediment sliding on the skin replicas

Experiments on skin replicas were performed to investigate the effect of scale shape on sediment interaction and exclude the influence of mucus that might alter adhesive forces among the species. Microscopy showed that the replication technique could reproduce the structure of the original fish skin (Fig. 6). In the mould of each species, exposed ctenial spines could be identified. However, it has to be considered that in replicas the lack of flexible underlying tissue, in which scales are embedded, could also potentially influence the skin interaction with the sediment. Additionally, the pressure during the application of the polymer could have deflected the scales from their initial position, which would lead to replicas, whose scales lie closer to the top of each other than in the original skin.

**Figure 8:**
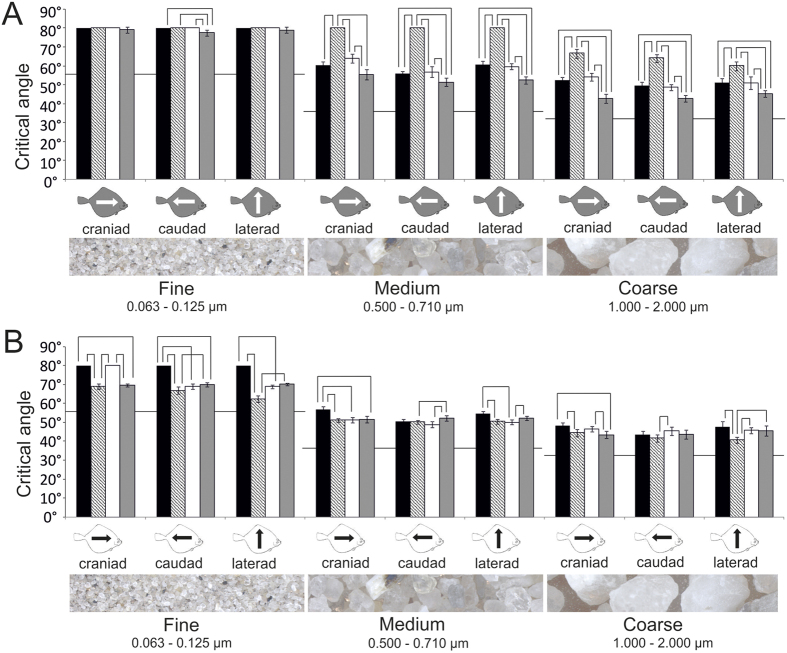
CSAs of the three sediment types (fine, medium, and coarse) in three directions: cranial, caudal, and lateral on resin replicas of the skin *S. solea* (black bars), *P. flesus* (hatched bars), *L. limanda* (white bars), *P. platessa* (gray bars) and on a smooth resin surface (black lines). (**A**) Replicas of the eye side. (**B**) Replicas of the blind side. Brackets indicates significant differences of CSAs of the different sliding directions (Kruskal-Wallis One-Way ANOVA on Ranks, P ≤ 0.001, Tukey test P < 0.05). Standard deviation is presented by error bars.

Significant differences between the eye and the blind side were found in all measurements except for the fine sediment in *S. solea* (all directions) and *L. limanda* (cranial direction) and the medium (caudal and lateral directions) and coarse sediments in *P. platessa* (all directions) (Fig. 7). At all replicas towards all directions, the CSA decreases significantly with higher grain size (Kruskal-Wallis One-Way ANOVA on ranks, 2 DF, P ≤ 0.001; pairwise multiple comparison Tukey test, P < 0.05). In comparison to the smooth resin surface, the CSA was higher for all skin replicas irrespective of both the sliding direction and sediment type (Fig. 8). However, also at the smooth surface, we observed a decrease of the CSA with an increasing grain size of the sediment (Fig. 8).

## Discussion

Our study found striking and statistically significant differences in scale shape and arrangement across the examined flatfish species and between the eye and the blind side of the individuals of the same species. All findings were in accordance to the descriptions of the taxonomic keys and comparative studies^1^,^2^,^3^,^19^. Our functional study revealed a high degree of correlation between morphological features of scales and their frictional properties in contact with sliding sediments. Although we could not exclude the influence of mucus completely, the direction- and grain-size-dependent differences in CSAs, as well as higher friction for the sediment of the medium grain on the eye side in all individuals studied, can be only interpreted as a result of different scale morphology between the eye side and the blind side. In *P. flesus,* whose scale density and scale morphology differed greatly on both sides of the body, we found significant differences in CSAs for all tested sediments. A similar effect was also obtained on skin replicas. The higher CSAs on the eye side for a wide range of grain sizes (0.125–2.000 μm) could be the results of (1) the presence of ctenoid scales mainly limited to the eye side and having a convex shape at the millimetre range and (2) the presence of vertical ctenial spines in the range of 100 μm. In *L. limanda* significant difference was found in all sliding directions, but only for the sediment with the grain sizes in the range of 0.500–0.710 μm. We found no significant differences in scale density and spine dimensions between both sides of the body, but the higher CSAs on the eye side are likely resulting from differences in the arrangement of scales within the tissue. Scales of the eye side are more exposed and overlap with each other more than those at the blind side. In accordance with the morphology, frictional properties at both body sides were the same for all grain sizes and sliding directions in both *S. solea* and *P. platessa*, having less difference in both the scale shape and arrangement across the body. As minor exception, *P. platessa* showed significant differences for the medium sediment in the cranial direction.

With the original skin, as well as on the skin replicas and on the smooth resin surface, the CSA decreases with an increasing grain size. Since the sand was placed as a very thin layer and therefore interactions within the sediment can be largely excluded as the reason for this effect, scaling effects resulting from the relationship between the contact area (scaling quadratically) and mass (scaling cubically) of the sand grains must lead to the observed effect. Deviating from this intrinsic grain size-dependent sliding behaviour observable on the smooth surface, in *S. solea* we found considerably higher CSAs for the medium grain size at both body sides than in the other species. The other three species showed for this sediment type the strongest differences between their eye and their blind side with significantly higher CSAs at the eye side. Irrespective whether the differences are a result of specific properties of the mucus or the scales morphology, these results demonstrate clearly the sediment-dependent species-specific differences which can be interpreted as an adaptation to certain sediments in the habitat, as well as an adaptation of scales of the eye side to increase the friction with sediments.

All in all, friction properties of skin replicas corresponded to that of original fish skin. The structure-based friction properties are thereby transferrable to other materials, although one could expect that the adhesive mucus, present in the intact skin, could mediate friction forces to a certain degree. In spite of the facts that replica scales were not embedded in a flexible underlying tissue and that they have different material properties from original scales, the interactions with sediments were quite similar in the original scales and their replicas. We conclude that overlapping scales do not spread out by external forces of the sediment leading to a higher roughness. Additionally, the polymer of the negative mould was pressed against the skin. The distance between overlapping scales is likely to be smaller in replicas than in the original scales. In fish having overlapping scales (*S. solea* and *L. limanda*) both artefacts lead to a decrease of surface roughness resulting in a decrease of friction on replicas.

In general, a rough surface topography, either macro- (exposed and convex scales) or microscopical (ctenial spines), seem to increase the CSA. Our experiment on the sediment sliding on the resin replicas of fish scales, showing higher CSAs than those on the smooth resin surface, as well as significantly higher CSAs at the rougher eye side, also demonstrate the advantage of rough scales in keeping sediment on their surface. Low CSAs on the unstructured cycloid scales on the eye side of *P. platessa,* observed for nearly all sediments and sliding directions and repeatable also in the measurements on skin replicas, show clearly that this scale type is less suited to generate high frictional forces which maintain the sand coverage. Conversely, ctenoid scales in *P. flesus,* limited to the eye side, and the larger number of spines found on scales on the eye side of *L. limanda* could be interpreted as an adaptation to keep sand on the relevant body side. In this case sediment interactions could be considered as one selective factor relevant to the evolution of pleuronectiform scale spines. Indeed, numerous studies describe selectivity and grain sizes dependent burying behaviour in Pleuronectiformes^4^,^5^,^6^,^7^, as well as a sediment-dependent abundance^6^,^9^. While the majority of studies on sediment dependency focuses on juveniles and nursery areas and are mainly based on data of pacific species^4^,^5^,^6^,^7^,^8^,^9^,^10^, very few studies address the role of sediment and other ecological factors for older individuals of the four species studied here. The analysis of the field data of the flatfish population in the North Irish Sea^20^ allows a direct comparison of the ecological niches. Although the classification of sediments by grain size was broader and beyond the range tested in our study, the analysis of Amezcua and Nash^20^ revealed notable differences in the sediment preference existing between the species. Despite different sediment classes, one can at least draw conclusions about a general trend, because the categories “gravel” and “mud” prolongate our sediment classes “fine” and “coarse” beyond the range of 0.062 mm-2 mm. In both fishing seasons analyzed (March and October) *L. limanda, P. platessa,* and *S. solea* showed high abundance on sand (grain size: 0.0625-2 mm), while their density varied seasonally on gravel (grain size: 2–256 mm), mud (grain size: <0.0625 mm), and mixed sediments (grain size: <256 mm) indicating that the species prefer sand, but spread out to other less attractive sediments at high abundance^20^. Nevertheless, *L. limanda* and *P. platessa* were less abundant on gravel, whereas *S. solea* showed the lowest abundance on mud (grain size: <0.0625 mm) during both periods^20^. Neither the species-specific scale morphology (the scale size and the presence and shape of cteni) nor the different sediment-dependent friction behavior of the fish skin, revealed in our study, could be explained by the patterns of sediment preference found for the species studied by Amezcua and Nash^20^.

Beside sediment interaction as a possible reason for the development of the high diversity of scale types in Pleuronectiformes, other scale properties could be taken into account.

Flatfish have an excellent camouflage, and have the ability to adapt the colour and pattern of their eye side to the optical appearance of the sea bottom^11^,^13^,^14^. The shape, texture, and arrangement of scale of the eye side are presumably adapted to the specific optical properties, scales of the blind side have more frequent contacts with the sea bottom and could have been more adapted to maintain friction with dense bulk sand or to reduce abrasive wear of the skin. Moreover, scales may have a protective function against predators and may reduce drag while swimming. The importance of adaptations depends on a variety of ecological factors, such as the visibility condition and predator pressure, as well as species specific properties, such as diurnal activity pattern or prey spectrum, and could also be potentially considered as selective factors influencing scale morphology.

On the basis of their food spectrum, flatfishes can also be grouped in feeder classes. A comprehensive review of the current literature on trophic ecology of flatfishes and their feeder classes is provided by Link, Fogarty and Langton^21^. The species studied here are polychaete and small crustacean feeders (all four species), echinoderm feeders (*L. limanda*), and siphon tip and benthos feeders (all except for *S. solea*). Among the large tested number of morphological traits in Pleuronectiformes (the volume cavity and the gape of the mouth, the width, length, and transient thrust factor of the body, as well as the distance and diameter of the eyes) only the mouth gape was found to correlate with the food spectrum^22^. The main diet of our species consists of oligochaetes, amphipods, chironomids, small harpacticoids, copepods, mysids, polychaetes, chironomids, clams, bivalves, and siphon tips^21^, which let expect less adaptations of the skin. However, for flatfish feeding on food animals with a well-developed visual system, a good camouflage by a thin layer of sediment, hold by high skin friction, may be advantageous. Whereas *P. flesus* and *L. limanda* are described to be also fish predators^22^,^23^,^24^ even at daytime^23^, *S. solea* and *P. platessa* do not eat significant amounts of fish^22^,^24^. However, this pattern of diet could neither explain the differences in scale morphology nor the species-specific friction behavior of the fish skin found in our study.

The present study is the first attempt to investigate the relationship between the scale morphology and their functional properties in Pleuronectiformes. Further comparative morphological and ecological studies are needed to shed light on the scale evolution in this fish group as well as knowledge of the flatfish behavior, such as the bury behaviour or the response to water currents, which has been described so far only semi-quantitatively and solely in individual species^25^,^26^,^27^,^28^,^29^. For instance, *P. platessa* was observed to show rheotactic response orienting its head in the direction of the current^27^, which could explain the asymmetrical scale morphology causing also the direction-dependent friction properties measured in our study.

Further functional studies could examine other functions of the scales. For instance the mechanical role of mucus in Pleuronectiformes remains unclear. For longer swimming distances, a sticky body surface keeping well sand particles might cause high drag and can be therefore disadvantageous. For this reason *S. solea* removes the sand by performing a specific movement, the omega-jump^26^. However, removal movements in Pleuronectiformes have never been investigated in a comparative study.

In general, functional aspects of the fish scale morphology were mainly studied in sharks due to drag-reducing properties^30^,^31^,^32^,^33^,^34^,^35^. However, the properties and functional aspects of the vast majority of other fish scales remain unknown. We hope that the present study will initiate such research activities, because this knowledge is very important not only for better understanding biology of species, but also for biomimetic as possible inspiration for synthesis of novel material with new interesting properties.

## Material and Methods

### Animals

Scale morphology and the critical sliding angle for sediments were investigated in four European flatfish species, the Common Sole, *Solea solea* (Linnaeus, 1758), the European Plaice, *Pleuronectes platessa* Linnaeus, 1758, the European Flounder, *Platichthys flesus*, (Linnaeus, 1758), and the Common Dab, *Limanda limanda* (Linnaeus, 1758). Three individuals of *P. flesus* and four individuals of each of the other species were obtained fresh from a commercial fish trader (Matjes Lange GmbH) and kept stored on ice. The length of the individuals was about 36.0 ± 2.1 cm in *S. solea*, 38.5 ± 1.5 cm in *P. platessa*, 33.0 cm ± 0.8 in *P. flesus*, and 31.8 ± 1.5 cm in *L. limanda*.

### Resin replicas

Experiments were also performed with replicas of the fish skin. Skin of the centre (close to the lateral line) of the eye side and the blind side was casted with a polymer Polyvinylsiloxane (PVS) (PRESIDENT Plus Jet™ light body l, coltène whaledent^®^, Langenau, Germany) and filled with epoxy resin^36^ (61.3% NSA, 23.6% ERL 4221, 14.2% D.E.R. 736, 0.9% DMAE). After a curing period of one day at 60 °C, the 10 × 10 cm large resin replicas were separated from the negative PVS moulds.

### Scale morphology

The density of scales was measured on 2 × 2 cm areas at the ventral and dorsal parts of the eye and blind sides of the fish. Four individuals per species were examined. A scale bar was placed on the fish and photographed with a camera (Panasonic Lumix DMC-FZ18). From the photos scales were counted. The overall length of each individual was also determined. Additionally, scale morphology was also investigated by using light and scanning electron microscopy.

For scanning electron microscopy (SEM), skin pieces of about 12 mm diameter were dissected from the eye side and the blind side of each fish 3–5 cm above and below the lateral line. The skin was fixed on aluminium stubs with double-sided carbon conductive tape (Plano, Wetzlar, Germany) and desiccated in a drying chamber filled with silica gel for 12 h. After this drying process the samples were coated with a 15 nm thick layer of palladium-gold using a sputtering device Leica EM SCD 500 (Leica, Wetzlar, Germany) and examined in a SEM Hitachi TM3000 (Hitachi High-Technologies Corp., Tokyo, Japan). For light microscopy, pieces with a diameter of approximately 10 cm of fresh skin from the eye side and the blind side 3–5 cm from the lateral line upward and downward, respectively, were examined under a stereo microscope Leica M205A (Leica, Wetzlar, Germany). Therefore, the pieces of the skin were placed in Petri dishes filled with fresh water and covered with a glass plate to prevent light reflections. The replica and the three sediments were examined with the same setup but under dry conditions. Ctenial spines were counted and measured from the images of the original skin, as well as from those of the replicas.

### Critical sliding angle of the sediment

The critical sliding angle of different sediment types was investigated on fish individuals placed in a transparent plastic box (20 × 30 cm) with sea water (Dupla Marin, Premium Sea Salt, 33 g/1 litre of fresh water) and fixed with pins at both the tail and the head on the bottom. A circular area of 3 cm diameter on the back of fishes was covered with a thin layer of one type of the sediment (fine, medium and coarse) in random order. The different sand layers had a weight of approximately 0,1 g (fine sediment), 0,2 g (medium sediment) and 0,4 g (coarse sediment). A plastic tube was used to place the sand on the fish skin to assure an identical volume and thickness of the sand layers for each sample.

The measurements were done as follows. The plastic box with the fish was placed on a metal plate. The plate was tilted by a string mounted to a motor until the critical angle was reached at which the sand slid down. Critical angles were measured by using a protractor. Each individual was tilted caudad, craniad, and laterad with the blind side and the eye side upwards in a random order. Skin replicas of the eye side and the blind side of the four species were measured in the same way. The fish and replicas were pinned on a polystyrene sheet which was fixed with a hot-melt glue applicator on the bottom of the box.

### Sediments

All experiments were conducted with grain-size controlled sediments. Quartz sand (SiO_2_) was obtained from a commercial dealer (Raab Karcher, Kiel, Germany) and sorted by sieves (Retsch GmbH, Haan, Germany) into three size classes according to the Wentworth scales (fine sand: 125–250 μm grain size; coarse sand: 500–1000 μm grain size; very coarse sand: 1–2 mm grain size). The particle size distribution of the three sediment classes was determined by dry-sieving analyses. The grain-size classes were determined according to 1/4 of Phi unit i.e from 63 μm to 2 mm (see supplementary materials: S1). The density of each sediment class was determined on the principle of Archimedes. 50 g of each sediment was poured in 50 ml water and the volume of the displaced water was measured. Afterwards the density ρ could be determined by the quotient of the sediments mass and the volume of water displaced by the sediment. The density of each sediment amounted to 2.63 g/cm^3^.

## Additional Information

**How to cite this article**: Spinner, M. *et al*. Key role of scale morphology in flatfishes (Pleuronectiformes) in the ability to keep sand. *Sci. Rep.* **6**, 26308; doi: 10.1038/srep26308 (2016).

## Supplementary Material

Supplementary Information
