# Supplementary material for: Key role of scale morphology in flatfishes (Pleuronectiformes) in the ability to keep sand
Source: Sci Rep. 2016 May 20;6:26308. doi: 10.1038/srep26308 (PMC4873799; doi:10.1038/srep26308)
Supplement: Supplementary Information [file srep26308-s1.pdf]

Key role of scale morphology in flatfishes (Pleuronectiformes) in the ability to keep sand

Marlene Spinner, Mareike Kortmann, Camille Traini, Stanislav N. Gorb

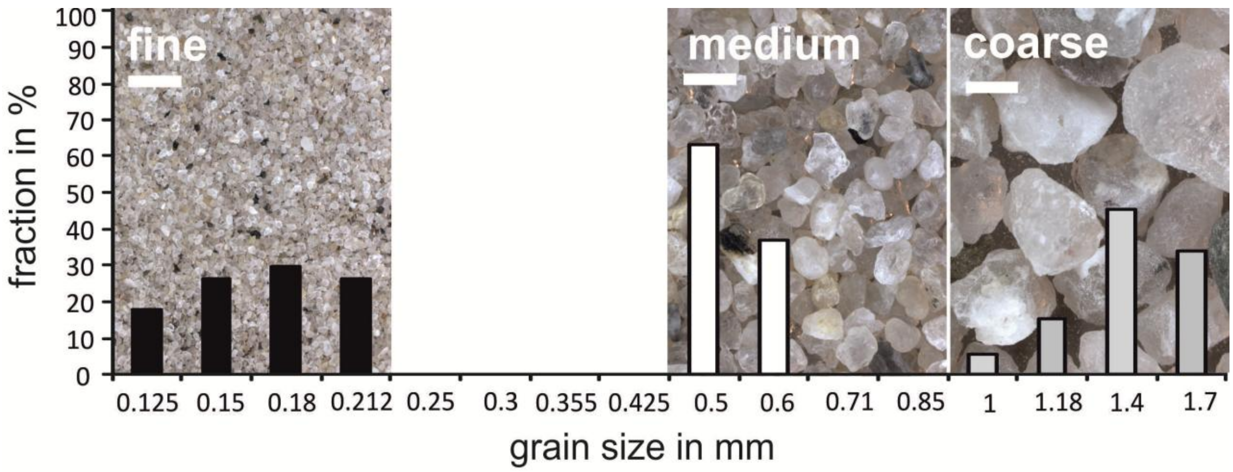

S1. Light microscopy images and distribution of grain sizes of the used sediments. Grain sizes (Wentworth classes): fine, coarse (here: medium), and very coarse sand (here: coarse) according to ISO 565 and ISO 3310 (R20/3 and R 40/3). The scale bars are 1 mm.

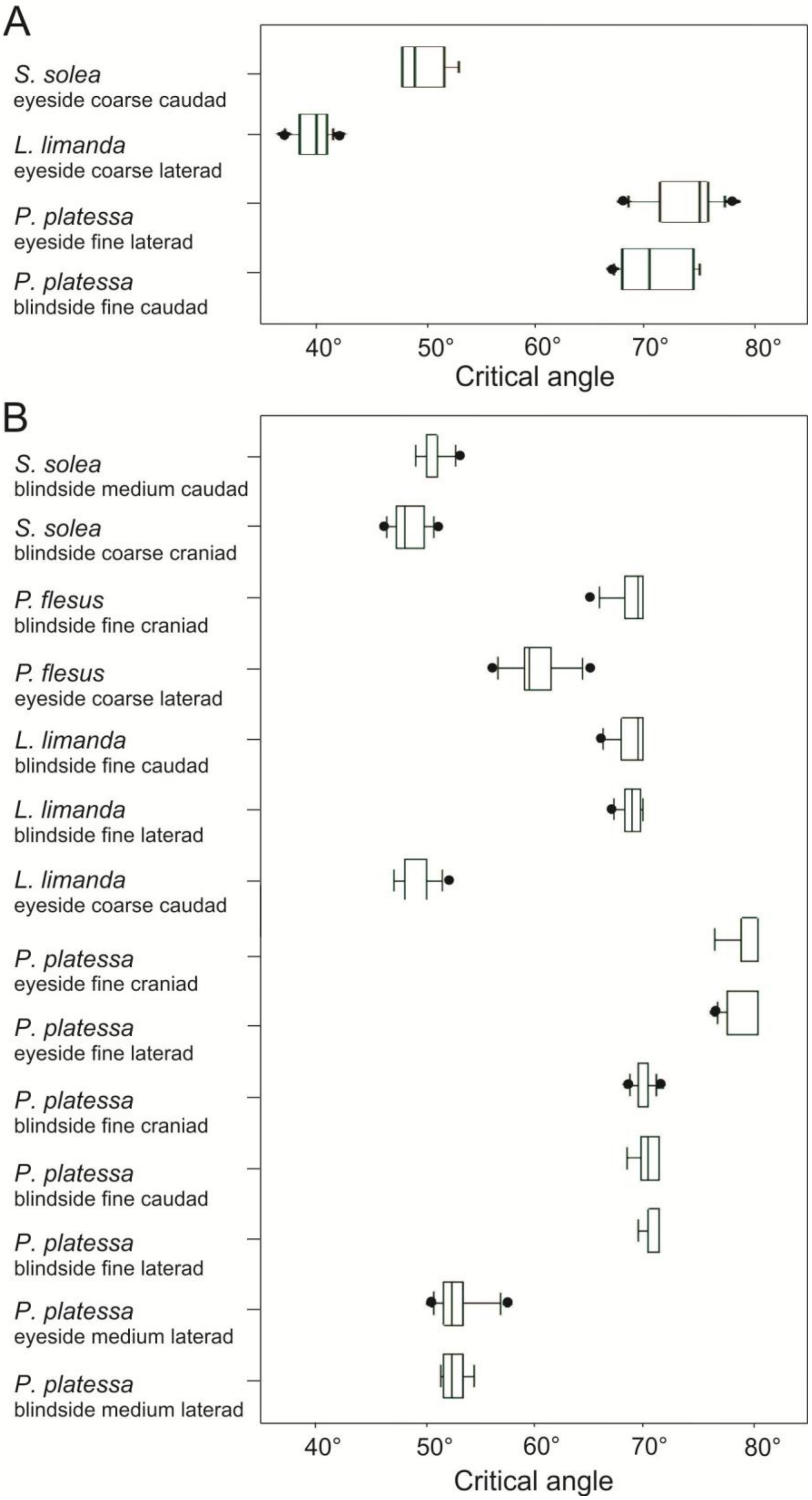

S2. Box plot of CSAs of the three sediment types (fine, medium, and coarse) on original skin and resin replicas of the skin of the eye side and blind side of *S. solea*, *P. flesus*, *L. limanda*, and *P. platessa* in three directions: cranial, caudal and lateral. (A) Original skin. (B) Resin replicas of the the skin. The median is presented by a vertical line, the boxes indicate the upper and lower quartile. Minimum and maximum values and outliers are presented by error bars and dots.

	fine		medium		coarse	
	ES	BS	ES	BS	ES	BS
S. solea						
craniad x caudad	-	-	-	-	5.384 *	6.672 *
craniad x laterad	-	-	-	-	6.453 *	5.165 *
caudad x laterad	-	-	-	-	1.069	1.507
H	-	-	-	-	24.168	24.623
P. flesus						
craniad x caudad	-	-	5.522 *	6.803 *	4.956 *	5.208 *
craniad x laterad	-	-	4.683 *	3.402 *	5.124 *	4.998*
caudad x laterad	-	-	0.840	3.402 *	0.168	0.210
H	-	-	18.619	23.249	16.997	17.491
L. limanda						
craniad x caudad	-	-	6.836 *	6.288 *	4.658 *	6.014 *
craniad x laterad	-	-	5.000 *	4.685 *	7.179 *	5.822 *
caudad x laterad	-	-	1.836	1.603	2.521	0.192
H	-	-	25.523	21.454	26.843	23.506
P. platessa						
craniad x caudad	3.699 *	4.850 *	6.233 *	6.096 *	4.631 *	5.740 *
craniad x laterad	6.658 *	5.137 *	5.603 *	5.740 *	7.206 *	6.096 *
caudad x laterad	2.959	0.288	0.630	0.356	2.576	0.356
H	24.422	17.846	23.712	23.487	26.761	23.533

S3. Table: Direction-dependent difference of CSAs of sediments (fine, medium, coarse) on the eye (ES) and blind sides (BS) of the examined flatfish species. H and q-values of Kruskal-Wallis One Way ANOVA on ranks (2 DF, $P \leq 0.001$) and a subsequent pairwise multiple comparison Tukey test ($P < 0.05$). Asterisks indicate significant differences.

	craniad		caudad		laterad	
	ES	BS	ES	BS	ES	BS
S. solea						
fine x medium	0.000	0.000	0.000	0.000	0.000	0.000
fine x coarse	5.918 *	5.918 *	5.918 *	5.918 *	5.918 *	5.918 *
medium x coarse	5.918 *	5.918 *	5.918 *	5.918 *	5.918 *	5.918 *
H	33.243	33.261	33.261	33.225	33.213	33.188
P. flesus						
fine x medium	0.000	3.402 *	3.402 *	3.444 *	3.402 *	3.402 *
fine x coarse	5.103 *	6.803 *	6.803 *	6.761 *	6.803 *	6.803 *
medium x coarse	5.103 *	3.402 *	3.402 *	3.318 *	3.402 *	3.402 *
H	24.658	24.029	24.076	23.820	24.067	24.061
L. limanda						
fine x medium	0.986	4.124 *	3.946 *	3.946 *	3.946 *	3.946 *
fine x coarse	6.412 *	7.713 *	7.891 *	7.891 *	7.891 *	7.891 *
medium x coarse	5.425 *	3.589 *	3.946 *	3.946 *	3.946 *	3.946 *
H	29.764	30.986	32.420	32.381	32.442	32.381
P. platessa						
fine x medium	3.946 *	3.946 *	3.946 *	4.137 *	3.973 *	4.274 *
fine x coarse	7.891 *	7.891 *	7.891 *	7.699 *	7.864 *	7.562 *
medium x coarse	3.946 *	3.946 *	3.946 *	3.562 *	3.891 *	3.288
H	32.355	32.093	31.300	29.772	31.056	28.861

S4, Table: Grain-size-dependent difference of CSAs of sediments on the eye side (ES) and blind side (BS) of the examined flatfish species. H and q-values of Kruskal-Wallis One Way ANOVA on ranks (2 DF, $P \leq 0.001$) and a subsequent pairwise multiple comparison Tukey test ($P < 0.05$). Asterisks indicate significant differences.
